# Kinesin Family of Proteins Kif11 and Kif21B Act as Inhibitory Constraints of Excitatory Synaptic Transmission Through Distinct Mechanisms

**DOI:** 10.1038/s41598-018-35634-7

**Published:** 2018-11-27

**Authors:** Supriya Swarnkar, Yosef Avchalumov, Bindu L. Raveendra, Eddie Grinman, Sathyanarayanan V. Puthanveettil

**Affiliations:** Department of Neuroscience, The Scripps Research Institute, Scripps Florida, 130 Scripps Way, Jupiter, Florida 33458 USA

## Abstract

Despite our understanding of the functions of the kinesin family of motor proteins (Kifs) in neurons, their specific roles in neuronal communication are less understood. To address this, by carrying out RNAi-mediated loss of function studies, we assessed the necessity of 18 Kifs in excitatory synaptic transmission in mouse primary hippocampal neurons prepared from both sexes. Our measurements of excitatory post-synaptic currents (EPSCs) have identified 7 Kifs that were found to be not critical and 11 Kifs that are essential for synaptic transmission by impacting either frequency or amplitude or both components of EPSCs. Intriguingly we found that knockdown of mitotic Kif4A and Kif11 and post-mitotic Kif21B resulted in an increase in EPSCs suggesting that they function as inhibitory constraints on synaptic transmission. Furthermore, Kifs (11, 21B, 13B) with distinct effects on synaptic transmission are expressed in the same hippocampal neuron. Mechanistically, unlike Kif21B, Kif11 requires the activity of pre-synaptic NMDARs. In addition, we find that Kif11 knockdown enhanced dendritic arborization, synapse number, expression of synaptic vesicle proteins synaptophysin and active zone protein Piccolo. Moreover, expression of Piccolo constrained Kif11 function in synaptic transmission. Together these results suggest that neurons are able to utilize specific Kifs as tools for calibrating synaptic function. These studies bring novel insights into the biology of Kifs and functioning of neural circuits.

## Introduction

Kinesin molecular motor proteins are ATPase dependent machines that move along the tracks formed by microtubules to facilitate the transport of cargo within the cell. Molecular motors possess different binding sites: (1) for the cargo to be transported and (2) for the interactions with tubulin subunits to produce mechanical shift. The rearrangement of tubulin subunits is the basic requirement during mitosis for the formation of mitotic spindle and kinesins play a critical role by promoting directionality for the movement^1^. Since its discovery in 1985^2,3^, several members of the kinesin family (Kif) have been identified^4,5^. Kifs are classified into 15 kinesin families, which are termed Kinesin 1 to Kinesin 14B^6,7^. Gene expression analyses have shown that most of the 45 Kif genes encoded by the mouse genome are expressed in cultured hippocampal neurons^8^.

Loss of function, imaging and biochemical analyses have identified critical roles for Kifs in cell division^9,10^, brain development^11^, learning and memory^12–16^, neurological disorders such as Alzheimer’s disease (AD)^17^, Amyotrophic lateral sclerosis (ALS)^18,19^, hereditary spastic paraplegia SPG10^20,21^ and neurodevelopmental disorders such as intellectual disability^22,23^. These multiple phenotypes could be attributed to two broad functions of Kifs: force generating and cytoskeleton regulating function of mitotic Kifs and cargo transporting functions of post-mitotic Kifs. During cell division, mitotic Kifs such as Kif11 facilitate movement of spindles, whereas post-mitotic such as Kif17 and Kif5C transport cargos from the cell body to synapses. Intriguingly, force generating Kifs are also important in post-mitotic neurons. For example, force generating Kifs such as Kif2A^24^ and Kif19A^25^ functions as microtubule-depolymerizing Kifs through distinct mechanisms in post-mitotic neurons.

Transported cargos include proteins such as Piccolo^12,26^, Neurexin^12,27^, Glutamate receptors^28,29^, organelles such as vesicles and mitochondria^30,31^ and RNAs such as CaMK21^13,17,32^ and BDNF^33^. Taken together these two broad functions suggest that Kifs might play a central role in regulating synapse function. Consistent with this idea a number of Kifs that are necessary for synaptic transmission were identified. For example, Willemsen *et al*.^23^ demonstrated that Kif4A and Kif5C are important in regulating excitatory as well as inhibitory synaptic transmission in hippocampal neurons. Kif4A knockdown resulted in a decrease in miniature inhibitory post-synaptic currents (mIPSCs), but an increase in miniature excitatory post-synaptic currents (mEPSCs). Nakajima *et al*.^34^ found that in Kif5A conditional knockout mice, the amplitude of mIPSCs was significantly reduced, whereas the frequency of mIPSCs was unaltered^34^. Taken together these studies suggested that Kifs have specific roles in synaptic transmission. However, it is not known whether only a subset or all Kifs are critical for synaptic transmission, and the mechanisms by which Kifs exert regulation of excitatory synaptic transmission. Furthermore, considering the large number of Kifs encoded in the mammalian genome, it is not known whether multiple Kifs that regulate synaptic transmission could be expressed in the same neuron.

To address this, we carried out electrophysiological analyses of synaptic transmission following knockdown of 18 different Kifs representing 6 different families. Intriguingly, we identified 11 Kifs that are critical for synaptic transmission whereas 7 Kifs do not have a significant effect in this process. Furthermore, we identified three Kifs that act as inhibitory constraints on synaptic transmission. Our mEPSC analysis of four Kifs (Kif13B, 21A, 21B and 11) identified Kifs that regulate synaptic transmission through post-synaptic, and/or pre-synaptic mechanisms. Importantly we found that Kifs that act pre- or post-synaptically could be expressed in the same neuron. Furthermore, we found that the activity of pre-synaptic NMDAR and expression of Piccolo are necessary for the effects of Kif11 on excitatory synaptic transmission.

## Results

### Identification of Kifs that are necessary for excitatory synaptic transmission

**Figure 1:**
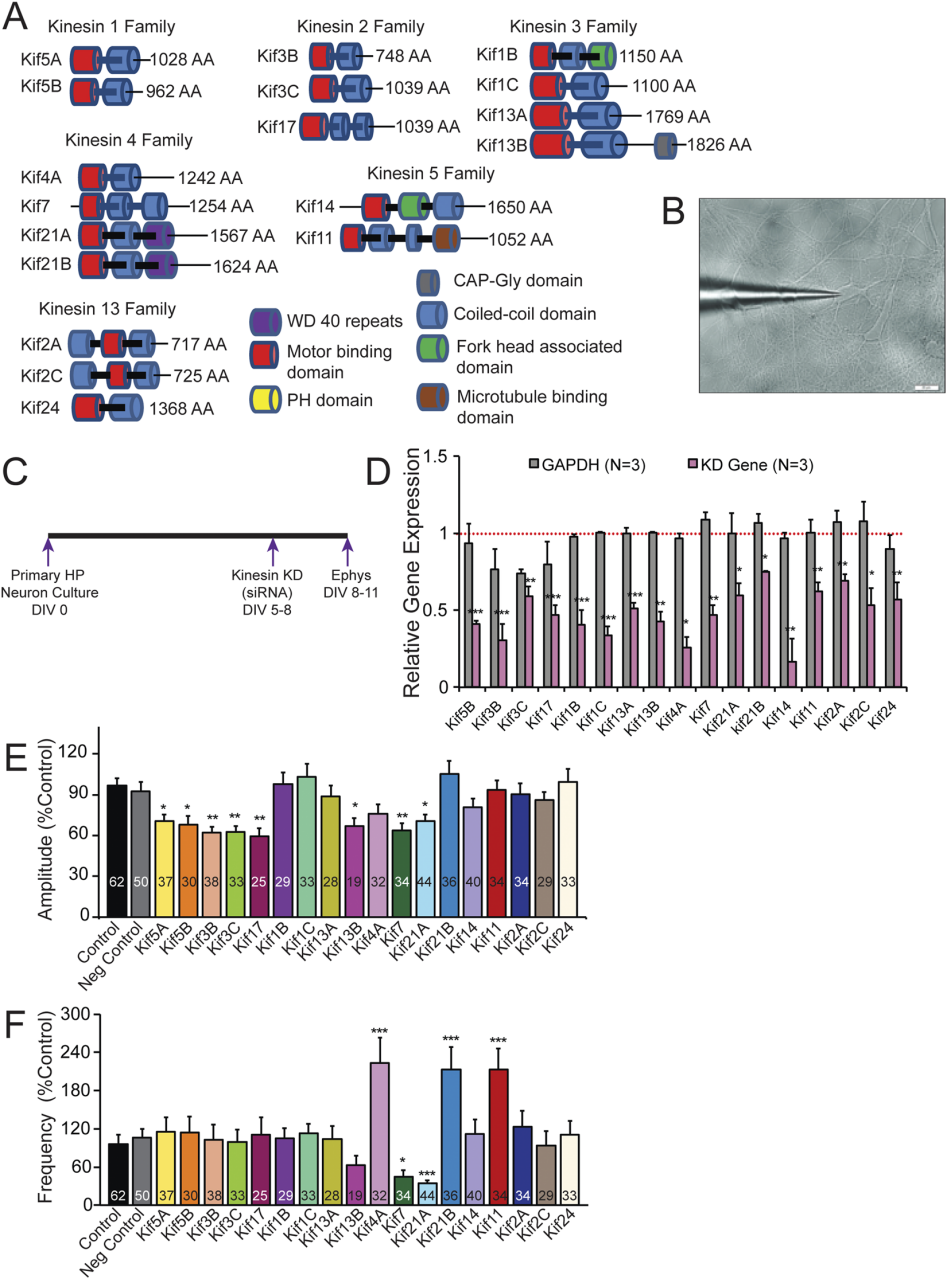
Identification of Kifs that are necessary for spontaneous excitatory synaptic transmission. (**A**) Cartoon showing domain organization 18 Kifs selected from 6 families. (**B**) Image showing patch clamp recording electrode attached to a neuron. (**C**) Schematic showing the experimental strategy to record spontaneous excitatory post-synaptic potentials (sEPSCs) in the mouse primary hippocampal neuron culture following knockdown (KD) of different Kifs. (**D**) Analysis of knockdown of Kifs by quantitative real time PCR (qPCR). Kifs were knocked down individually, isolated RNAs and analyzed expression of corresponding Kifs along with GAPDH for comparisons. Data was normalized to 18srRNA levels. Bar graphs show relative expression of Kifs following knockdown of individual Kifs in hippocampal neurons 72 hrs. after transfection of siRNAs. A nontargeting siRNA was used as specificity control. Error bars are SEM, Student’s *t*-test, *p-value < 0.05. (**E**,**F**) Bar graphs showing amplitudes and frequencies of sEPSCs respectively following 72 hrs. knockdown of Kifs. The number of neurons patched in each group labeled in bar graphs. Data (mean) from all the knockdown groups compared to negative control (non-targeting siRNA). Also see Supplementary Fig. S1 and Additional File Table S1. *p < 0.01, One-Way ANOVA followed by Dunnett’s post hoc test. Error bars are SEM.

To assess whether different Kifs play a unique role in synaptic transmission, we selected 18 Kifs representing 6 Kif families (Fig. 1A; Kinesin 1, 2, 3, 4, 5 & 13 families) based on Hirokawa *et al*.^6^ and examined their role in excitatory synaptic transmission in hippocampal neurons. These Kifs include those that are involved in transporting cargos (Kif 5A & 5B, 17, 1B & C, 13A & B, 14, 21A & B, 24) and those involved in microtubule rearrangements (Kif 2A & C, 3B & C, 4A, 7, 11). Importantly Kif 2A, 2 C, 4A & 11 are mitotic Kifs involved in spindle movement.

To evaluate the role of the 18 Kifs in synaptic transmission, we carried out RNAi mediated loss of function studies (Fig. 1B,C). Using siRNAs we first knocked down Kifs individually in primary hippocampal neurons. A nontargeting siRNA was used as a control along with mock-transfected neurons. Quantitative real-time PCR (qPCR) analysis of Kif knockdowns by respective siRNAs (Fig. 1D) shows efficient knockdown of these Kifs within 72 hrs. of transfection of siRNAs (Additional File Table S1) in hippocampal neurons. We next measured spontaneous excitatory post-synaptic currents (sEPSCs) by whole cell patch clamp recordings following knockdown of individual Kifs. sEPSCS are currents generated by action-potential-dependent as well as –independent release of neurotransmitter in the absence of experimental stimulation. Briefly, following the knockdown of individual Kifs using siRNAs, we measured sEPSCs in DIV 8–11 neurons and analyzed the amplitude and frequency components (Fig. 1E,F; Additional File Table S1). Representative sEPSC traces are shown in Supplementary Fig. S1. sEPSC measurements (Fig. 1E,F) identified Kifs whose knockdown do not affect amplitude (10 Kifs: 1B, 1C, 13A, 4A, 21B, 14, 11, 2A, 2C, 24) or frequency (13Kifs: 5A, 5B, 3B, 3C, 17, 1B, 1C, 13A, 13B, 14, 2A, 2C, 24) or both (7Kifs 1B, 1C, 13A, 14, 2A, 2C, 24). Similarly, Kifs whose knockdown produced a significant change only in frequency (3Kifs: Kif4A, 21B, Kif11) and only in amplitude (6Kifs: 5A, 5B, 3B, 3C, 17, 13B) and both amplitude and frequency components (2Kifs: 7, 21A) were identified. Consistent with these results, cumulative probability analysis of sEPSC measurements show corresponding differences in amplitude and frequency (Supplementary Fig. S1).

Intriguingly, knockdown of mitotic kinesins Kif11 and 4A and transporting kinesin Kif21B produced an increase in the frequency of sEPSCs suggesting that these Kifs might function as inhibitory constraints on synaptic transmission. Taken together these results have identified Kifs that are nonessential for synaptic transmission and Kifs that are positive and negative regulators of excitatory synaptic transmission.

### Elucidating the pre-and post-synaptic role of Kifs in mediating synaptic transmission

**Figure 2:**
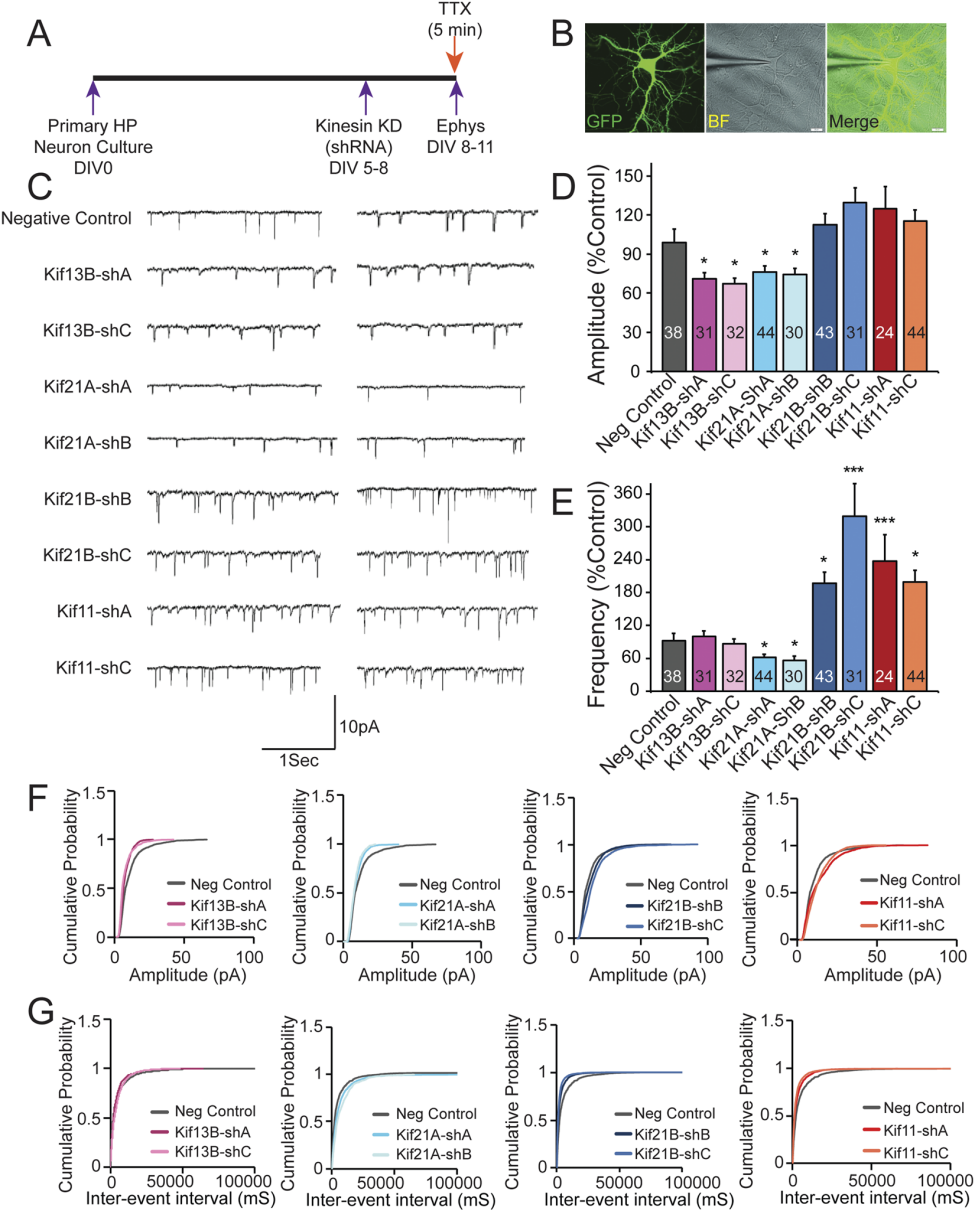
Identification of pre- and post-synaptic roles of Kifs in synaptic transmission. (**A**) Schematics showing the experimental strategy to record miniature excitatory post-synaptic potential (mEPSCs) in the mouse primary hippocampal neuronal cultures following knockdown of Kifs using shRNAs. (**B**) Image showing eGFP labeled neuron in fluorescence and bright field (BF) with patch clamp recording electrode attached. (**C**) Two representative traces of mEPSCs for control (no shRNA), negative control (scrambled shRNA) and shRNAs against specific Kifs. (**D**,**E**) Bar graphs showing changes in amplitudes and frequencies of mEPSCs respectively following 72 hrs. knockdown of specific Kifs. The number of neurons patched in each group is labeled in the bar graph. Data (mean) from all the knockdown groups compared to negative control. *p < 0.05, one-way ANOVA followed by Dunnett’s post hoc test. Error bars are SEM. Also see Additional File Table S2 for values used in the bar graphs. (**F**,**G**) Cumulative probability graph showing changes in amplitude and frequency respectively of mEPSCs between control, control shRNA and Kif shRNA (Kolmogorov-Smirnov Test, p < 0.05).

To further understand the modulation of synaptic transmission by Kifs, we focused on four Kifs (negative regulators: 21B, Kif11; positive regulators: 13B and 21A) and sought to determine whether they affect synaptic transmission through pre-synaptic, post-synaptic or both mechanisms. Hence, we measured miniature EPSCs (mEPSCs) (Fig. 2) by recording EPSCs in the presence of tetradotoxin (TTX). mEPSCs are pre-synaptic action potential independent unlike sEPSCs and are thought to correspond to the response that is elicited by a single vesicle of neurotransmitter. Therefore, a change in the amplitude corresponds to post-synaptic change whereas a change in the frequency corresponds to a pre-synaptic change.

We used two independent shRNAs (shRNA A/B/C) to knockdown each of these four Kifs (Fig. 2). A nontargeting shRNA transfected neurons were used as control. shRNA plasmids also express eGFP for visualizing the neuron. mEPSC analyses show no changes in the amplitude with knockdown of Kif21B and Kif11, but significant changes in amplitude with Kif13B and Kif21A knockdown (Fig. 2D). Similarly, analyses of mEPSC frequencies (Fig. 2E) show an increase in frequency with 21B and Kif11 knockdown, whereas a decrease in frequency with Kif21A knockdown, and no effect on frequency with Kif13B knockdown (Additional File Table S2, one-way ANOVA, Dunnett’s post-hoc test, *p < 0.05). The cumulative probability analysis of the data shows corresponding differences in amplitude and frequency for each Kif (Fig. 2F,G).

Together these results suggest that Kif21B and Kif11 function through pre-synaptic mechanisms, whereas Kif13B impact synaptic transmission through post-synaptic mechanism and Kif21A act through both pre- and post-synaptic mechanisms. Increased frequency in mEPSCs by Kif21B and Kif11 indicate changes in pre-synaptic release probability.

### Kif11, Kif21b and Kif13b are expressed in the same hippocampal neuron

**Figure 3:**
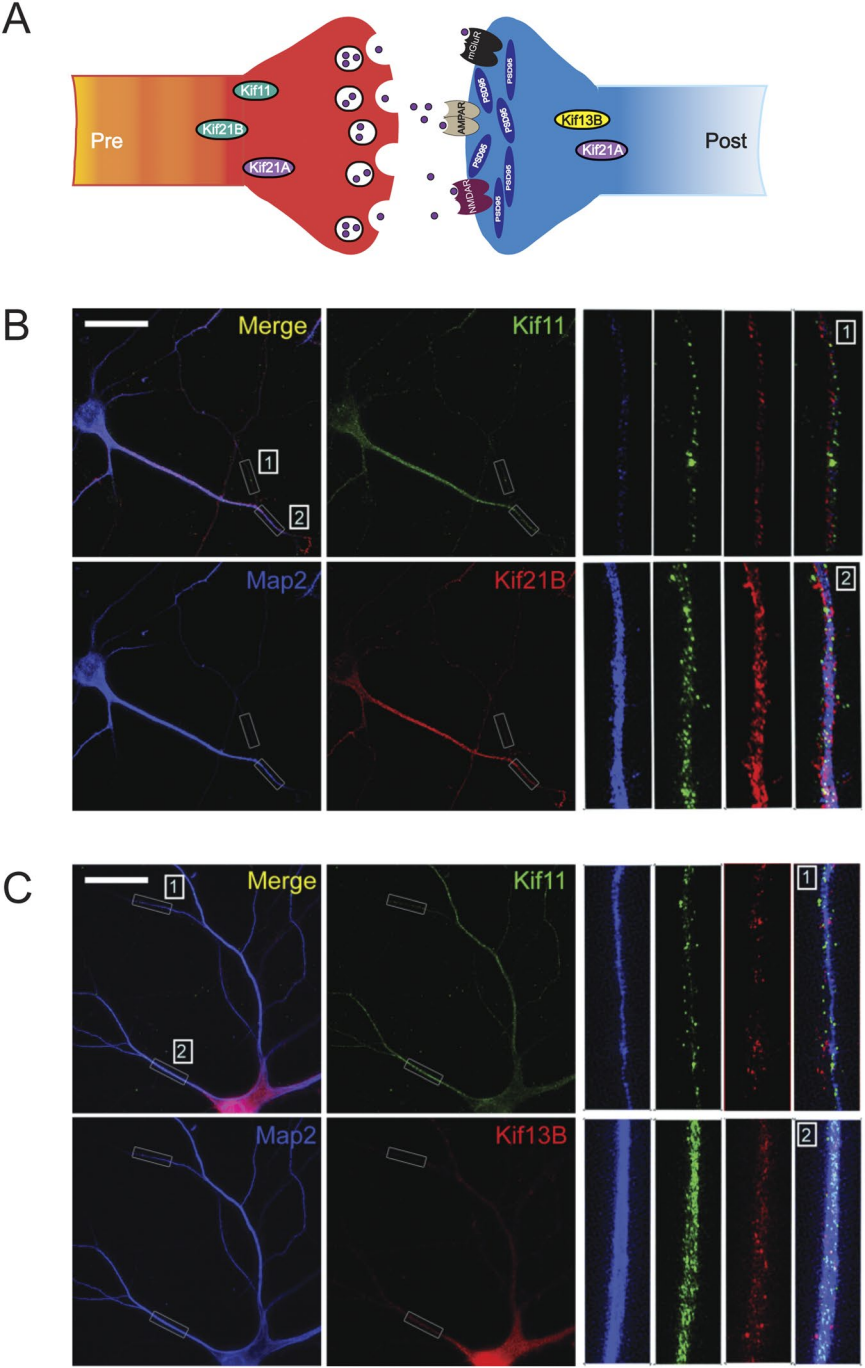
Kifs that function pre- or post-synaptically are expressed in the same hippocampal neuron. (**A**) Summary of the electrophysiological analysis of mEPSCs showing pre- and post-synaptic role of Kif21A, 21B, 13B and 11. (**B**) Representative super-resolution images showing the distribution of Kif11 (green) and Kif21B (red) proteins as puncta in the same hippocampal neuron. Digitally enlarged inset images (corresponding to Kif11, Kif21B, Map2 and merged). (**C**) Representative super- resolution images showing the distribution of Kif11 (green) and Kif13B (red) proteins in the same hippocampal neuron. Digitally enlarged inset images (corresponding to Kif11, Kif13B, Map2 and merged). The Map2 (blue) staining indicates the dendritic localization of Kifs. Shown are two representative sections from neurites marked 1 and 2 (in white). Scale bar: 20 μm.

Our finding that Kifs regulate synaptic transmission through distinct mechanisms led us to ask whether Kifs that function pre- or post-synaptically are expressed in different or in the same neurons. Expression of these Kifs in the same neuron would suggest unique ways by which the neuron can employ Kifs for calibrating synapse function. Our electrophysiology data suggested that 21B and Kif11 act pre-synaptically, 13B acts post-synaptically and Kif21A acts both pre-and post-synaptically (Fig. 3A). Because Kif11, 13B and 21B functions either pre- or post-synaptically, we next asked whether these Kifs are expressed in the same hippocampal neurons. To address this we carried out super-resolution structured illumination microscopy (SIM) imaging.

We performed double labeling experiments to detect expression of either Kif11 and 21B together or Kif11 and 13B together. Our SIM imaging shows that these kinesins are expressed as distinct punctate structures with Kif13B abundant in the neuronal cell body. All three kinesins are expressed in the distal processes. SIM images shown in Fig. 3B,C indicate that Kif11 and 21B are expressed in the same neuron and Kif11 and 13B are expressed in the same neuron. Together, these results suggest that Kifs with a unique mechanism of regulation of synaptic transmission are expressed in the same neurons.

**Figure 4:**
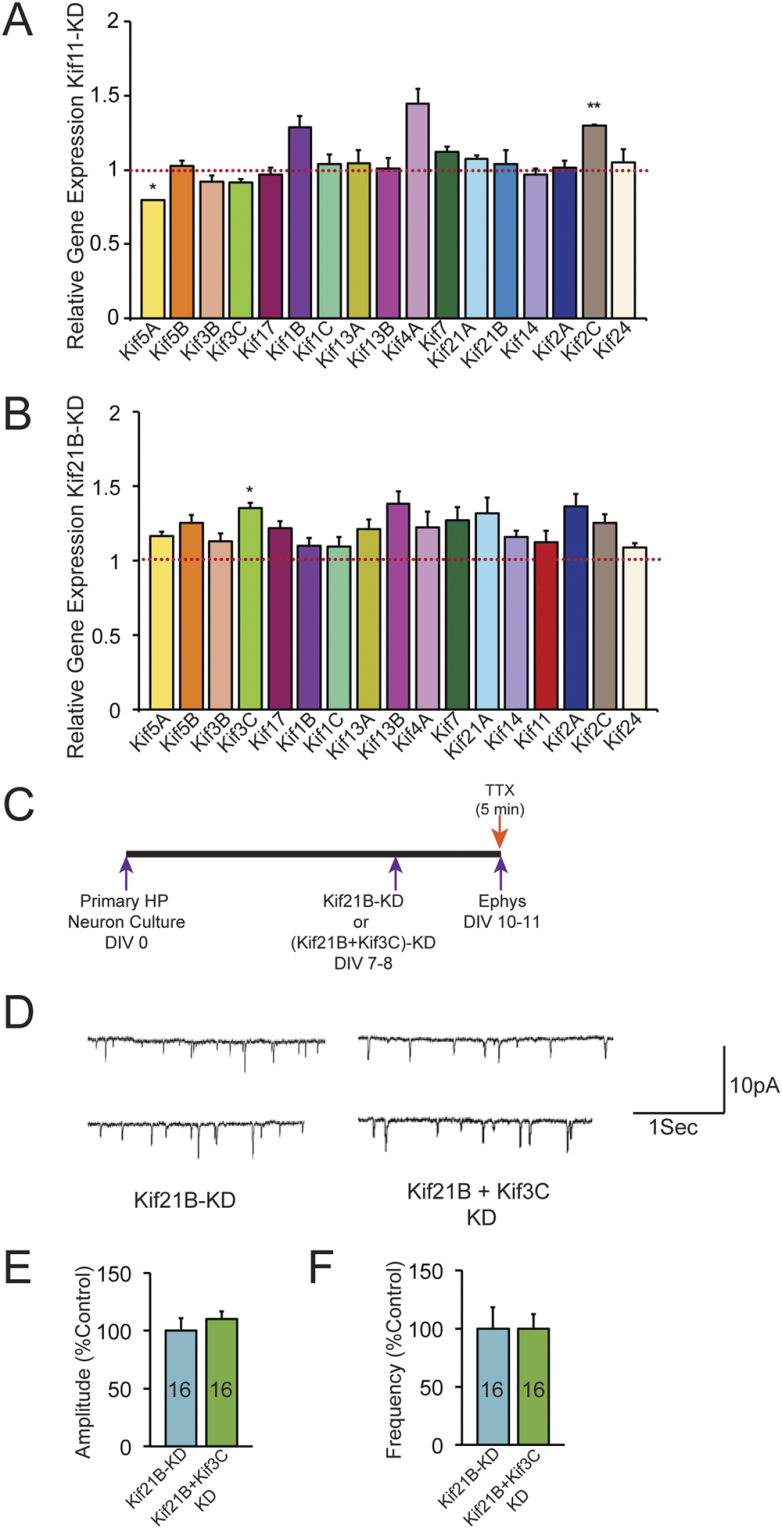
Effect of Kif11 and Kif21B knockdown on expression of other Kifs. (**A**,**B**) qPCR analyses of relative expression changes in 17 Kifs, in Kif11 and Kif21B knockdown neurons respectively after 72 hrs. of transfection of Kif11 or Kif21B siRNA compared to non-targeting siRNA control in hippocampal neurons. Data was normalized to 18srRNA levels. Error bars are SEM, *p < 0.05, Student’s *t* test. (**C**) Schematics showing the experimental strategy to record mEPSCs in the mouse primary hippocampal neuronal cultures after Kif knockdowns (KD). (**D**) Two representative traces of mEPSCs recorded from Kif21B KD and double knockdown (Kif21B + Kif3C KD) hippocampal neurons. (**E**,**F**) Bar graphs show mEPSC amplitude and frequency respectively of Kif21B KD and Kif21B + Kif3C KD neurons. Numbers of neurons patched in each group are labeled in the bar graphs. Error bars are SEM. Student’s *t* test, p = 0.42. Also see Additional File Table S3 for values used in the bar graphs.

To gain insight into the molecular basis of enhanced synaptic transmission with Kif11 and Kif21B knockdown, we considered the possibility that knockdown of the Kif11 and 21B might result in the recruitment of specific Kifs resulting in increased transport and enhanced synaptic transmission. Therefore, we assessed the mRNA expression of 18 Kifs following Kif11 or Kif21B knockdown using siRNAs (Fig. 4A,B, Additional File Table S3). qPCR analysis shows that Kif11 knockdown resulted in significant increase in Kif2C expression and a decrease in expression of Kif5A whereas expressions of 15 other Kifs were not significantly affected. Similarly, knockdown of Kif21B resulted in increased mRNA levels of Kif3C, whereas expressions of 16 others Kifs were unaltered. Taken together the qPCR analysis of Kif knockdowns identified specific changes in the expression of other Kifs.

To comprehend the significance of upregulation of Kif2C and Kif3C, we carried out single-cell qPCR experiments in which, using a patch pipette, we aspirated cellular contents for RNA analysis. We extracted RNAs from 10 eGFP labeled neurons following knockdown of Kif11 or Kif21B using shRNAs and assessed expression of Kif2C or Kif3C by qPCRs. The expression levels of Kif2C mRNA levels were found to be too low in these cells (ct values >30, n = 2, 10 neurons each) in Kif11 knockdown neurons that we could not confirm whether Kif2C mRNAs were indeed upregulated with Kif11 knockdown in eGFP labeled neurons. It could be that the starting levels Kif2C were too low in the single hippocampal neuron. Although there might be an increase in Kif2C mRNAs with Kif11 knockdown, qPCR analysis of 10 neurons might not be sufficient to detect a change in Kif2C levels. The knockdown experiment described in Fig. 1 used >500,000 neurons (5 × 10^5^ neurons/sample) and siRNAs were used to knockdown Kif11, which has higher transfection efficiency compared to shRNAs. Hence Kif11 is knocked down in a larger number of neurons compared to shRNAs and we could detect specific differences in Kif2C following Kif11 knockdown by siRNAs.

We carried out similar single-cell qPCR experiments with Kif21B knockdown and confirmed upregulation of Kif3C mRNAs (~2-fold upregulation, 20 neurons each, Supplementary Fig. S2) with Kif21B knockdown. We then asked whether Kif3C might mediate KIf21B effects on synaptic transmission (Fig. 4C–F). We knocked down Kif21B using shRNAs and Kif3C using siRNAs and measured mEPSCs. Non-targeting siRNAs were used as a control. A decrease in mEPSC would suggest a role for Kif3C in mediating Kif21B effects. Our mEPSC measurements in Fig. 4E,F show that Kif3C knockdown did not affect the increase in the frequency of mEPSCs in Kif21B knockdown neurons (Additional File Table S3). There was no change in the amplitude of mEPSCs when Kif21B and Kif3C double knockdown neurons (110.34 ± 6.39%, n = 16) as compared to Kif21B alone knockdown (100 ± 10.88%, n = 16, Student’s *t* test, p = 0.41). Also, there was no change in the frequency of mEPSCs in Kif21B and Kif3C double knockdown neurons (99.68 ± 12.38%, n = 16) as compared to Kif21B alone knockdown (100 ± 18.21%, n = 16, Student’s *t*-test, p = 0.98). Taken together these results suggest that Kif3C does not mediate the effects of Kif21B on synaptic transmission.

### Kif11 knockdown enhance activity of pre-synaptic NMDARs

We next sought to determine pre-synaptic mechanisms that underlie Kif11 and Kif21B effects on synaptic transmission. Kif11 is a mitotic Kif involved in chromosome positioning^35^, and establishing bipolar spindle during mitosis^36^. In post-mitotic neurons, Kif11 acts as a break for microtubule polymerization^37^. Kif21B is processive Kif that is enriched at microtubule plus ends and induces inhibition of growth of microtubule plus ends^38^. To understand the enhancement in synaptic transmission with Kif11 or Kif21B knockdown, we searched for pre-synaptic mechanisms that enhance synaptic transmission. Several studies have shown that pre-synaptic NMDAR (preNMDAR) mediate enhanced synaptic transmission^39,40^. Therefore, we then considered the possibility that preNMDARs mediate Kif11 and/or Kif21B effects on synaptic transmission.

**Figure 5:**
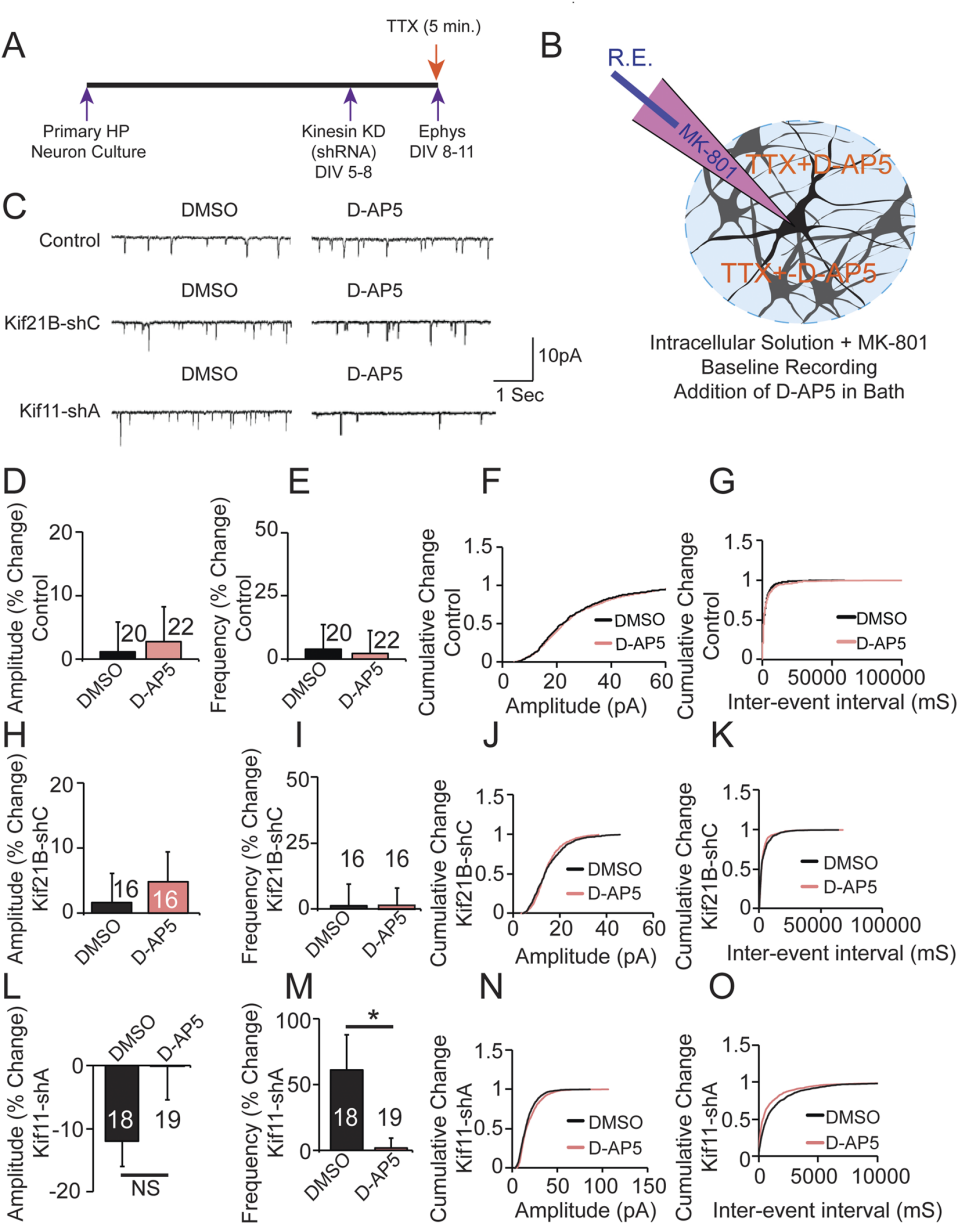
Pre-synaptic NMDARs are required for the Kif11 effects on synaptic transmission. (**A**) Schematics showing the experimental strategy to record mEPSCs in the mouse primary hippocampal neuron to determine pre-synaptic mechanisms of Kif11 on synaptic transmission. (**B**) Cartoon illustrating treatments and recording (R.E) electrode (filled with MK-801). (**C**) Representative traces of mEPSCs of Control, Kif21B and Kif11 knockdown in the presence of DMSO or D-AP5. (**D**,**E**) Bar graphs showing mEPSC amplitude and frequency respectively in control neurons in the presence of DMSO or D-AP5 (Student’s *t* test, p < 0.05). (**F**,**G**) Cumulative probability graphs showing mEPSC amplitudes and frequencies respectively of control neurons in the presence of DMSO or D-AP5 (Kolmogorov-Smirnov Test, p > 0.05). (**H**,**I**) Bar graphs showing mEPSC amplitude and frequency respectively following Kif21B knockdown in the presence of DMSO or D-AP5 (Student’s *t* test, p < 0.05). (**J**,**K**) Cumulative probability graphs showing mEPSC amplitudes and frequencies respectively of Kif21B knockdown neurons in the presence of DMSO or D-AP5 (Kolmogorov-Smirnov Test, p > 0.05). (**L**,**M**) Bar graphs showing mEPSC amplitude (p = 0.08) and frequency (p = 0.033) respectively in Kif11 knockdown neurons in the presence of DMSO or D- AP5 (Student’s *t* test). Also see Additional File Table S4 for values used in the bar graphs. (**N**,**O**) Cumulative probability graphs showing changes in mEPSC amplitudes and frequencies respectively of Kif11 knockdown neurons in the presence of DMSO or D-AP5 (Kolmogorov-Smirnov Test, for amplitude: p > 0.05; for frequency: p < 0.0000001). Numbers of neurons patched in each group are labeled in bar graphs. Data (Mean) from all the groups and error bars are SEM.

To test this idea, we used MK801 (an inhibitor of NMDAR) in the patch pipette to inhibit post-synaptic NMDA measurements and added APV/D-AP5 (an inhibitor of NMDAR) to the bath to block preNMDARs and recorded mEPSCs (Fig. 5A,B). DMSO (vehicle) was used as a control. Following the knockdown of Kif21B using shRNAs, we inhibited preNMDARs and measured mEPSCs in the presence of D-AP5 or DMSO in the bath. We observed no changes in amplitude or frequency of mEPSCs in the presence of D-AP5 compared to DMSO (Fig. 5H–K) similar to non targeting GFP alone transfected control neurons (Fig. 5D–G) suggesting that preNMDARs are not involved in the Kif21B knockdown induced changes in synaptic transmission. We next knocked down Kif11 and asked whether preNMDARs might mediate its effects. Our mEPSC measurements show no significant changes in the amplitude, however, we found that D-AP5 blocked the increase in the frequency of mEPSC due to Kif11 knockdown (Fig. 5L–O) suggesting that activity of preNMDARs is a critical component of the inhibitory constraint imposed by Kif11 on synaptic transmission. The change in amplitude with Kif11 knockdown and D-AP5 application was −0.06 ± 5.325%, n = 19 as compared to DMSO −11.99 ± 3.90%, n = 18; (Student’s *t* test, p = 0.08, Additional File Table S4). However, changes in frequency in the presence of D-AP5 when we knockdown Kif11 1.93 ± 7.73%, n = 19 in comparison to DMSO application 61.33 ± 26.27%, n = 18 (Student’s *t* test, p = 0.033, Additional File Table S4) were significant. Cumulative probability analysis show corresponding changes in amplitude and frequency of mEPSCs. Taken together, these results suggest that Kif11 knockdown results in the recruitment of preNMDARs for enhancing synaptic transmission.

### Kif11 knockdown increases dendritic arborization without changing spine density

**Figure 6:**
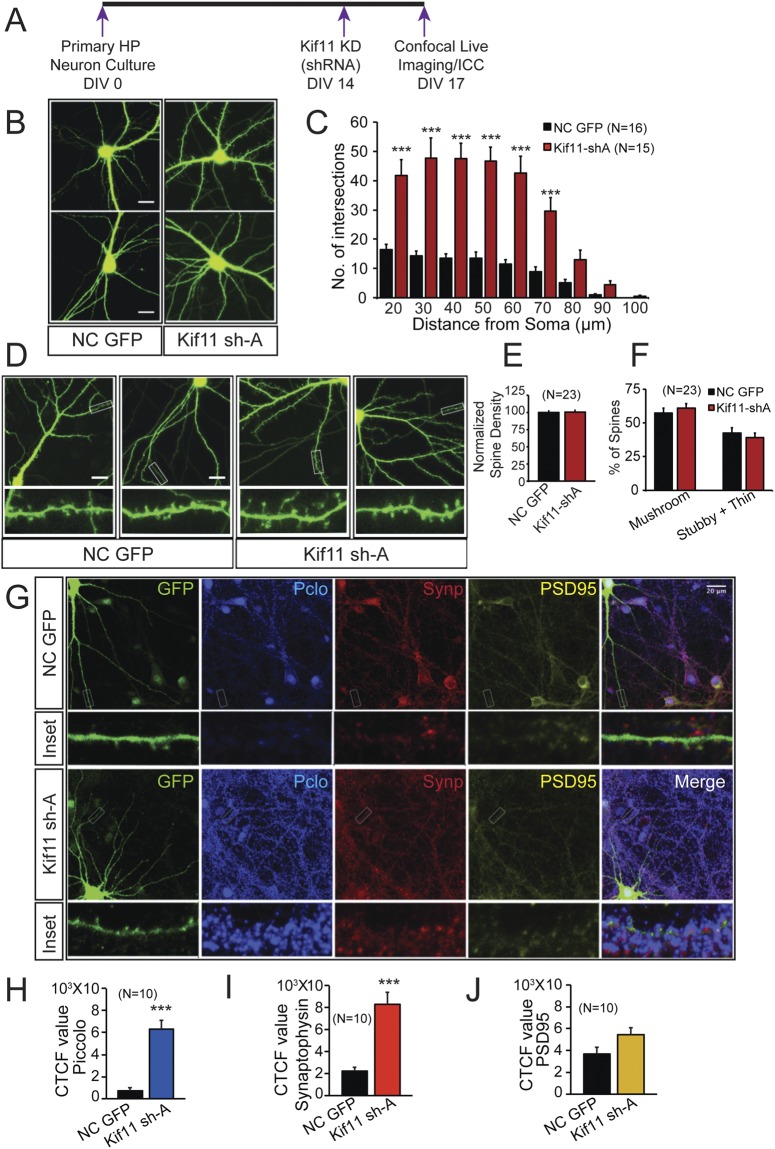
Kif11 knockdown produces specific changes in neuronal morphology and localization of synaptic proteins. (**A**) Schematics showing the experimental strategy in the mouse primary hippocampal neuron culture. (**B**) Representative confocal projection images showing soma in the center to depict the dendritic arbor. (**C**) Bar graphs showing the quantification of dendritic morphology changes in terms of number of intersections per 10 μm step size, by Sholl analysis (using Image J). (**D**) Representative confocal projection images show no changes in spine, digitally enlarged image in inset. (**E**) Quantitative analyses of image data shown in “D” were carried out using MATLAB software selecting 100 μm length of dendrites. Bar graphs show no change in spine density or in spine morphology (**F**). (**G**) Representative confocal projection images and digitally enlarged insets images show the expression of Piccolo (blue), Synaptophysin (red) and PSD-95 (yellow) puncta in eGFP (green) labeled dendrites along with merge images. (**H**,**I**,**J**) Bar graph show relative corrected total cell fluorescence (CTCF) values corresponding to fluorescence intensity of Piccolo, Synaptophysin and PSD- 95 respectively analyzed using NIH image J. Changes were compared between Kif11 knockdown and control (scrambled shRNA). Student’s *t* test, error bars are SEM. ***p < 0.0005. Scale bar 20 μm. Also see Additional File Table S5 for values used in the bar graphs.

To understand the mechanism underlying the increase in the synaptic transmission with Kif11 knockdown we next asked whether Kif11 knockdown produces any morphological changes in neurons. Sustained enhancements in synaptic transmission could be resulting from an increase in the number of functional synapses or specific changes in the pre-synaptic release machinery. Previous studies^41,42^ using superior cervical ganglia (SCG) neurons (DIV5-6), have shown that inhibition of Kif11 enhanced dendritic arborization. However, whether Kif11 knockdown also affects spine number and whether Kif11 has a similar effect on dendritic arborization in hippocampal neurons are not known. To address this, we used older primary hippocampal cultures (DIV17) that were transfected with a specific shRNA to knockdown Kif11 or control shRNA (on DIV14). Confocal live cell imaging of DIV17 hippocampal neurons was used to assess whether dendritic and spine changes are produced with Kif11 knockdown (Fig. 6A–F).

Sholl analysis of confocal images (Fig. 6B,C) showed increased dendritic arborization from 20–70 μm distance from soma (Additional File Table S5, one-way ANOVA Tukey post hoc test, ***p < 0.0001) in Kif11 knockdown hippocampal neurons compared to neurons that are transfected with nontargeting shRNA control. These results are consistent with the previous findings on Kif11 effects on dendritic arborization in SCG neurons. We then quantified spine density per 100 μm dendrites and assessed changes in spine morphology (Fig. 6D–F). We found that Kif11 knockdown did not produce a change in spine density (Kif11 shA 100.5 ± 2.6 and Control 100 ± 2.5, N = 23) or spine morphology (Mushroom spines: Kif11 shA 61.01 ± 3.37, Control 57.4 ± 3.4, and stubby or thin spines: Kif11 shA 38.98 ± 3.6, Control 42.6 ± 3.7, N = 23, Fig. 6, Additional File Table S5). Although there was no change in the spine density, because we observed an overall increase in dendritic arborization, we conclude that Kif11 knockdown increases total spine number in hippocampal neurons. This change in spine number might underlie the enhanced synaptic transmission with Kif11 knockdown.

To assess whether Kif11 impacts presynaptic machinery for synaptic transmission, we imaged two pre-synaptic proteins (Piccolo and Synaptophysin) and a post-synaptic protein (PSD95). Piccolo is an active zone protein^43^ and Synaptophysin is a key protein involved in vesicle endocytosis^44^, whereas PSD95 is required for the post-synaptic regulation of synaptic transmission^45^. PSD95 expression levels are also used as a measure of changes in spine density^46^. We next carried out immunocytochemical imaging of Piccolo, Synaptophysin and PSD95 on DIV17 hippocampal neurons (Fig. 6G–J). A non-targeting shRNA plasmid was used for comparing the effect of Kif11 knockdown. Confocal imaging analyses showed an increase in Piccolo (6314.2 ± 805.5 as compared to control 705.7 ± 294.8, N = 10, Student’s *t* test, p < 0.0001) and Synaptophysin (8303.6 ± 1082.8 as compared to control 2230.2 ± 370.8, N = 10, Student’s *t* test, ***p < 0.0001) CTCF value evaluated from fluorescence intensity of puncta, however there was no change in the PSD95 (5430.4 ± 652 as compared to control 3681.2 ± 629.4, N = 10, Student’s *t* test, p = 0.07, Additional File Table S5) expression with Kif11 knockdown. Because we average the immunocytochemical data per unit length of dendrites, as in the case of spine number, we conclude that Kif11 knockdown results in an overall increase in spine number and PSD95 puncta, although there was no specific change in the PSD95 expression per unit dendritic length analyzed. Taken together, these results suggest that while there is an overall increase in spine number and PSD95, Kif11 knockdown produce specific enhancements in expression of pre-synaptic components of excitatory synaptic transmission.

### Expression of Piccolo constrain the Kif11 effects on synaptic transmission

**Figure 7:**
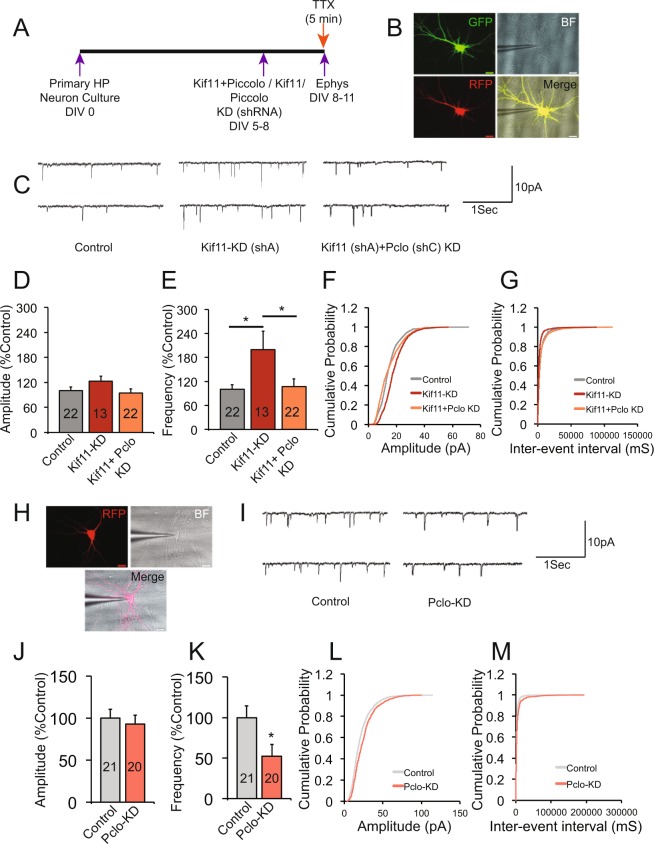
Expression of Piccolo constrains Kif11 effects on synaptic transmission. (**A**) Schematics showing the experimental strategy to record mEPSCs to assess the role of the Piccolo. (**B**) Image showing dual-labeled (eGFP and RFP) neuron in fluorescence and bright field with patch clamp recording electrode attached. (**C**) Two representative mEPSCs traces of control, Kif11KD and Kif11 + Piccolo knockdown (KD) recorded from mouse pyramidal neurons. (**D**,**E**) Bar graph showing changes in mEPSC amplitude (p = 0.1825) and frequency (p = 0.015) respectively show that expression of the Piccolo is required for the Kif11 effects on synaptic transmission (one-way ANOVA). (**F**,**G**) Cumulative probability graphs showing changes in amplitude and frequency respectively of mEPSCs (Kolmogorov-Smirnov Test, for amplitude: p > 0.05; for frequency: p < 0.000001). (**H**) Image showing RFP labeled neuron in fluorescence and bright field with patch clamp recording electrode attached. (**I**) Two representative traces of mEPSCs of control, Piccolo KD groups. (**J**,**K**) Bar graphs showing changes in the mEPSC amplitude (p = 0.628) and frequency (p = 0.024) respectively of control and Piccolo KD (shC) showing the effect of Piccolo KD on synaptic transmission (Student’s *t* test). Also see Additional File Table S6 for values used in the bar graphs. (**L**,**M**) Cumulative probability graphs showing changes in amplitude and frequency respectively of mEPSCs of control, and Piccolo KD (Kolmogorov-Smirnov Test, for amplitude: p > 0.05; for frequency: p < 0.000001). The number of neurons patched in each group labeled in the bar graph. Data (mean) from all the groups and error bars are ±SEM.

Because we found enhanced levels of Piccolo with Kif11 knockdown, we asked whether Piccolo can mediate the effects of Kif11 on synaptic transmission. If Piccolo expression is necessary for KIf11 effects, we would observe a decrease in synaptic transmission in neurons in which both Piccolo and Kif11 are knocked down. We first identified specific shRNA for efficient knockdown of Piccolo in hippocampal neurons (Supplementary Fig. S3A–C). Quantitative analysis of immunocytochemistry images shows that Piccolo shRNA B & C produced ~50% decrease in Piccolo levels in hippocampal neurons Piccolo (Pclo) ShB 512 ± 71.5, Pclo ShC 343 ± 51.3 as compared to control 1299-9 ± 216.8, N = 8, Additional File Table S5, one-way ANOVA, Tukey post hoc test, *p < 0.01). We next carried out knockdown of Kif11 using eGFP tagged shRNA (Kif11shA-eGFP) and RFP tagged shRNA for Piccolo (Piccolo shC-RFP) in the same hippocampal neurons (Fig. 7A–G) and measured mEPSCs. Consistent with a critical role for Piccolo in mediating the Kif11 effect on synaptic transmission, we found a significant decrease in the frequency of mEPSCs with Piccolo knockdown. Furthermore, there were no changes in the amplitude of mEPSCs when we synergistically knockdown Kif11 and Piccolo 94.61 ± 9.51%, n = 22 as compared to Kif11 alone knockdown 122.63 ± 11.91%, n = 13 or control 100 ± 9.29%, n = 22 (Additional File Table S6, one-way ANOVA Tukey post-hoc test, p = 0.1825). Interestingly, there was a significant decrease in frequency when we synergistically knockdown Kif11 and Piccolo 106.99 ± 18.9%, n = 22 as compared to Kif11 knockdown 199.63 ± 45.83%, n = 13. The cumulative probability analysis of mEPSC amplitude and frequency corresponded to specific changes in the amplitude and frequency (Fig. 7F,G).

We next knocked down Piccolo in hippocampal neurons and asked whether Piccolo expression is required for synaptic transmission (Fig. 7H–M). Our mEPSC measurements (Fig. 7J–M) show a specific effect on the frequency of mEPSCs (Additional File Table S6), Piccolo knockdown 52.57 ± 14.28%, n = 20 as compared to control 100 ± 14.25%, n = 21 (Student’s *t* test, *p < 0.05); without any changes in the amplitude Piccolo knockdown 92.80 ± 10.69%, n = 20 as compared to control 100 ± 10.19% (Student’s *t* test, p = 0.628) suggesting that post-developmental knockdown of Piccolo produces a presynaptic deficit in synaptic transmission. Taken together these results indicate that expression of Piccolo is required for the enhanced synaptic transmission due to Kif11 knockdown.

## Discussion

In this study, we assessed the role of 18 Kifs in excitatory synaptic communication. We found that Kifs can function as a positive or negative regulator of synaptic transmission and that not all Kifs have a critical role in synaptic transmission.

### Pre- vs post-synaptic functions of Kifs

Identification of key regulators of synaptic transmission would help elucidate the molecular underpinnings of neuronal communication and neural circuit function. Kifs might regulate neuronal communication by actively transporting cargos critical for synapse function or by regulating microtubules that provide tracks for active transport and forms integral components of the neuronal cytoskeleton. Focusing on 18 Kifs, we first asked three questions: whether (1) these Kifs are critical for synapse transmission, (2) their effects are restricted to specific components of synaptic transmission (pre- or post-synaptic), and (3) could the same neuron express Kifs that have a critical pre- or post-synaptic role?

To our surprise, sEPSC measurements following RNAi mediated loss of function suggested that not all Kifs expressed in neurons are essential for synaptic transmission. We identified 7 Kifs whose knockdown did not affect synaptic transmission. These Kifs include those that are mitotic (Kifs 2A^47^, Kif 2C^48^ and Kif24^49^) and those that have cargo transport function (Kif1B^50^, Kif1C^51^, and Kif13A^52^). It could be that some other Kifs could compensate their function or the extent of RNAi mediated knockdown might not be sufficient to produce a phenotype, although we observed significant knockdown. The 11 Kifs that we found to be critical for synaptic transmission affected specific components of sEPSC (3 Kifs: only frequency, 6 Kifs: only amplitude, and 2 Kifs: both amplitude and frequency), suggesting specific ways by which Kifs regulate spontaneous excitatory synaptic transmission.

To further understand the pre- or post-synaptic role of Kifs, we focused on four Kifs (Kif11, Kif21B, Kif13B, Kif21A) and carried out mEPSC measurements. We found that consistent with the sEPSC data, mEPSC measurements suggest that Kif11 and Kif21B function pre-synaptically and Kif13B post-synaptically and Kif21A act both pre- and post-synaptically. Taken together, these results suggest that Kifs modulate synaptic transmission in unique ways.

These findings led us to further explore whether Kifs (that specifically regulate pre- or post-synaptic components) could be expressed in the same neuron. Intriguingly, our super-resolution SIM imaging showed that these Kifs (pre-synaptic: Kif11 and Kif21B; post-synaptic: Kif13B) are expressed in the same neuron, suggesting the possibility that neurons express and use multiple Kifs in specific ways for calibrating synapse function. Consistent with this idea, Arpag *et al*.^53^ and Prevo *et al*.^54^ showed that multiple types of Kifs differentially regulate transport of the same cargo. However it is not known whether Kif11, 21B and 13B regulate localization of a specific cargo. Previously we have reported that^29^ Kif5C and Kif3A possess unique sets of protein cargos and are expressed in the same hippocampal neurons. Expressing multiple Kifs, each with specific effects on synaptic transmission and transport, will be advantageous to a neuron because these Kifs could be utilized for imparting specificity by differentially recruiting Kifs for regulating synapse function during development and plasticity.

### Pre-synaptic inhibitory constraints on excitatory synaptic transmission

sEPSC measurements suggested that knockdown of mitotic Kifs (Kif11 and 4A) and cargo transporting Kif (Kif21B) produced enhancements in synaptic transmission, suggesting that they function as inhibitory constraints on synaptic transmission. Among these three Kifs, we focused on two Kifs (11 and 21B) and found by mEPSC measurements that Kif11 and Kif21B act presynaptically in regulating excitatory synaptic transmission. Our SIM imaging show that Kif11 and Kif21B are expressed in the same neuron, suggesting that multiple inhibitory constraints could be expressed in the same neuron. We therefore searched for underlying mechanisms.

We considered the possibility that knockdown of these two Kifs might result in the recruitment of specific Kifs resulting in the enhanced transport of cargos for positively regulating synaptic transmission. By examining the expression of 17 Kifs following knockdown of these two Kifs in hippocampal neurons, we found that expression of specific Kifs (Kif2C with Kif11 knockdown and Kif3C with Kif21B knockdown) are indeed enhanced suggesting that Kif2C and Kif3C expression might mediate enhanced synaptic transmission associated with Kif11 and Kif21B knockdown respectively. Because, Kif2C knockdown by itself did not produce any effect on sEPSCs and we were unable to verify Kif2C knockdown in single neurons in which Kif11 was also knocked down, we could not conclude whether enhanced Kif2C expression is essential for Kif11 effects on synaptic transmission. We next assessed the significance of enhanced expression of Kif3C in mediating Kif21B effects by knocking down Kif3C in Kif21B knockdown neurons and measuring mEPSCs. We find that enhanced expression of Kif3C in Kif21B knockdown neurons is not critical for mediating Kif21B function in synaptic transmission, suggesting that Kif21B effects are independent of Kif3C expression changes.

Kif21A and 21B are homologous kinesins but differentially compartmentalized to axons and dendrites^55^. Kif21A is expressed both in axons and dendrites whereas Kif21B is enriched in dendrites. The axonal and dendritic expression might suggest pre- or post-synaptic functions because axons provide presynaptic terminals and dendrites, post-synaptic terminals. However, we find that Kif21A had both pre- and post-synaptic roles in synaptic transmission whereas Kif21B had a presynaptic role in regulating synaptic transmission, suggesting that their sub-cellular localization does not correlate to their roles in synaptic transmission.

Kif21B functions as a microtubule pausing factor^38^. Kif21B’s role in synaptic transmission was previously reported by Muhia *et al*.^16^, where they found that neurons from Kif21B knockout mice showed a decrease in the frequency of EPSCs, contrary to the increase in mEPSC frequency with shRNA mediated knockdown of Kif21B in hippocampal neurons. This inconsistency might be explained by the fact that in the knockout mice, a lack of Kif21B from the beginning of development might have recruited compensatory factors resulting in an altered phenotype, whereas in the post-developmental neurons, partial knockdown of Kif21B shRNA showed a different phenotype. In support of this, microtubules grew (regulation of microtubule dynamics is a key function of Kif21B) slower in Kif21B knockout neurons^16^ whereas in Kif21B shRNA mediated knocked down neurons, microtubules grew faster^56^.

We next focused on the pre-synaptic role of Kif11 and Kif21B and assessed whether preNMDAR might mediate enhanced synaptic transmission produced their knockdown. The role of preNMDARs in excitatory synaptic transmission has been demonstrated, however, it is not known whether preNMDARs mediate pre-synaptic effects of Kif11 or Kif21B. We found that unlike the case of Kif21B, Kif11 effects on synaptic transmission require the activity of preNMDARs.

Kif11 acts as a microtubule break and regulates axonal growth^57,58^. These studies showed that neurons, in which Kif11 is depleted, grow 5-fold longer and have more branches. Consistent with these findings, our experiments observed an increase in dendritic branching with Kif11 knockdown. We further concluded that Kif11 effects are not restricted to branching as there was an overall increase in spine number with Kif11 knockdown. The overall increase in spine density might underlie enhanced synaptic transmission. We then examined the expression of two critical components of synaptic transmission in the presynaptic compartment, synaptophysin and Piccolo. Imaging analyses showed an enhancement in Synaptophysin and Piccolo, suggesting changes in presynaptic machinery for synaptic transmission following Kif11 knockdown. We then assessed the role of Piccolo by RNAi mediated loss function and found that Kif11 effects on synaptic transmission require Piccolo. Thus, the activity of pre-NMDARs and Piccolo constrains effects of Kif11 on synaptic transmission. It remains to be determined how the microtubule breaking property of Kif11 is leading to the sustained enhancements in branching and spine density as well in the expression of presynaptic proteins Synaptophysin and Piccolo.

In summary, these results illuminate specific mechanisms by which neurons utilize Kifs for calibrating synaptic function. Presumably, these Kifs could be regulated in unique ways for adding specificity to synaptic function and/or to the structural changes associated with long-term synaptic plasticity. Identifying the molecular underpinnings of the functions of these Kifs, and when and how neurons utilize these Kifs are essential to our understanding of synapse biology and neural circuit plasticity.

## Materials and Methods

### Animals

Pregnant CD1 mice (Charles River Laboratories) were housed singly on a 12 h: 12 h light dark cycle with *ad libitum* access to food and water. All experiments were performed during the light part of the diurnal cycle. Housing, animal care and experimental procedures were consistent with the Guide for the Care and Use of Laboratory Animals and approved by the Institutional Animal Care and Use Committee of The Scripps Research Institute.

### Neuronal cultures, transfection of siRNAs and shRNAs

Primary hippocampal cultures were prepared from the brains of embryonic day 17–18 mice of both sexes. Cells were plated at a density of 5 × 10^5^ on poly-D-lysine-coated (500 μg/ml in RNase free water) dishes and 1 × 10^5^ glass coverslips. Cultures were plated in Neurobasal medium (Invitrogen) supplemented with 10% fetal bovine serum and penicillin/streptomycin mix and grown in Neurobasal medium supplemented with 2% B27 (Invitrogen), 0.5 mM glutamine, and penicillin/streptomycin mix at 37 °C in 5% CO_2_. Mouse SMARTpool siRNA reagent against all the Kifs was obtained from Dharmacon GE Healthcare. Mouse, 4 unique 29mer shRNA constructs in eGFP or RFP vector obtained from OriGene (TG501174, TG501180, TG501181, TG517925, TR30013, and TF502633). siRNA/shRNA were introduced to primary hippocampal neurons (5–8 days *in vitro* (DIV)) using Lipofectamine RNAiMAX or Lipofectamine 2000 (Invitrogen) according to manufacturer’s guidelines. Two different shRNAs were used to assess the effects of Kif knockdowns. One day before transfection, the fresh culture medium was prepared and mixed evenly with the old medium. One-half of the mixed mediums were left with the cells for transfection, and the other one-half was saved for medium replacement after transfection. For all our electrophysiological and imaging analyses, we performed our measurements on pyramidal neurons.

### Measurements of spontaneous and miniature excitatory post-synaptic currents (sEPSCs and mEPSCs)

Coverslips with cultured hippocampal neurons (DIV 8–11) were transferred to the perfusion chamber of an upright microscope and perfused with Extracellular bath solution (EBS) containing (in mM): 135 NaCl, 10 glucose, 3 CaCl_2_, 2 KCl, 2 MgCl_2_, and 5 Hepes, pH adjusted to 7.3–7.4 with NaOH, and 300–315 mOsm with Sucrose. Recordings were done to measure sEPSCs and mEPSCs under voltage clamp. Whole-cell patch-clamp recordings were performed using an Axon Multiclamp 700 b amplifier, 1440A Digidata digitizer and pClamp software (Axon Instruments, Foster City, CA). Recordings were made at 50 kHz and subsequently filtered at 5 kHz. The recordings were conducted blindly, and 22 groups were used during these experiments; Control, non-targeting Control-siRNA and Kif specific siRNA, Hippocampal neurons were cultured for at least 8 to 10 days to allow an extensive synaptic network to develop before recordings were made. The mEPSCs was recorded in the presence of TTX at 1 µM. The membrane potential was held at −60 mV during the recording of sEPSCs as well as during the recording of mEPSCs. The current clamp was recorded only to identify the health of the neurons. Only neurons with a resting membrane potential of less than −40 mV were used for our analysis. Action potentials were recorded in current clamp mode, I = 0. Membrane potential was maintained at least −40 mV in current clamp mode. All experiments were performed at room temperature (22–24 °C). The frequency and amplitude of EPSCs were analyzed using the template match search (pClamp) and basic statistical analysis performed to extract average amplitude and frequency. The amplitude and frequency of EPSCs expressed as a percentage of baseline level, calculated from an average of 5 min of the baseline-recording period. The amplitude and the frequency of EPSCs for each experiment were measured as the average of 5 min during the recording period. The shown ‘n’ values refer to the number of neurons recorded. Results are presented as mean ± SEM throughout the text unless otherwise noted. The term significant denotes a relationship with p < 0.05 determined using an unpaired Student’s *t* test, and one-way ANOVA with Dunnett’s or Tukey post hoc test. For the analysis of the cumulative probability of the amplitude and the cumulative probability of inter-event intervals, two-sample Smirnov-Kolmogorov test (SK test) was taken into the analysis, and difference was considered significant when p < 0.05.

### Measurement of pre-synaptic NMDA receptors

To measure pre-synaptic miniature (mEPSCs) of NMDA activity, neurons were hyperpolarized at −80 mV and MK-801 at 1 mM was added into the post-synaptic patch pipette, which nearly abolishes post-synaptic NMDAR activity^59^. Then the subsequent effects of bath application of NMDA receptor antagonist D-AP5 will reveal preNMDARs on neurotransmitter release. To measure preNMDARs on synaptic function, we measured mEPSCs amplitude and frequency before and after bath application of 50 µM of D-AP5. Interleaved control experiments were performed for the same duration in the presence of DMSO without D-AP5. The baseline period of mEPSCs was recorded for 2 min, and after 2 min of baseline recording, DMSO or D-AP5 were applied directly into the bath. Post-baseline was recorded for an additional 5 to 10 min. mESPCs were detected using an automatic template detection program and verified manually (pCLAMP; Molecular Devices^60^). All statistical analysis comparison was made between DMSO versus D-AP5 application, and Student’s *t* test was used for comparison between two groups, and statistical significance was defined as p < 0.05.

### Quantitative PCR (qPCR)

qPCR was carried out as described previously^29,61^. Sequences of the primers used in this study are shown in Additional File Table S8. Quantification of each transcript was normalized to the mouse 18S reference gene following the 2−ΔΔCt method^62^. Student’s *t* test was used to select genes with statistically significant expression levels where *p-value < 0.05.

### Sholl and Spine analysis

After 72 hours of transfection of hippocampal neurons using shRNA plasmid expressing eGFP, images of dendrites were collected at room temperature in the light microscopy facility at the Max Planck Florida Institute, using a confocal microscope (LSM 780; Carl Zeiss; Plan Neofluor 63X/1.3 N.A. Korr differential interference contrast M27 objective in water). Z-stack images were acquired using ZEN 2015 (64 bit) software (Carl Zeiss) and dendritic arbors were manually traced using confocal projection images and later quantified by Sholl analysis FIJI (ImageJ, NIH). The center of soma is considered as the midpoint and origin of the concentric radii was set from that point to the longest axis of soma. The parameters set for analysis were: starting radius 20 μm, ending radius 100 μm, radius step size 10 μm. The maximum value of sampled intersections reflecting the highest number of processes/branches in the arbor was calculated and the number of intersections plotted against distance from the soma center in μm. Data was analyzed using one-way ANOVA with Tukey post hoc test.

Spine morphology was analyzed using MATLAB software developed in the light microscopy facility at the Max Planck Florida Institute. By using a geometric approach, this software automatically detects and quantifies the structure of dendritic spines from the selected secondary branch (100 μm length) in the Z-stack confocal image. The software assigns the detected spines to one of the three morphological categories (thin, stubby or mushroom) based on the difference in structural components of the spines i.e. head, neck and shaft. Student’s *t* test was carried out to evaluate the statistical difference amongst the groups.

### Immunocytochemistry analysis

After 72 hrs. of transfection of primary neurons (on glass coverslips) with Kif11 shRNA or nontargeting control shRNA, neurons were processed for immunocytochemistry. Neuronal culture medium was carefully removed and, after two rinses in PBS, the cells were fixed in a freshly prepared solution of 4% paraformaldehyde for 15 min. After three or more rinses in PBS, the cells were permeabilized in 0.5% Triton X-100 in PBS for 15 min. The cells were then incubated in 8% normal goat serum in PBS for 45 minutes to reduce the non-specific binding of primary antibody followed by overnight incubation at 4 °C with primary antibodies: anti-Kif11 (1:1000, #ab51976, Abcam), anti-Synaptophysin (1:1000, #ab32594, Abcam), anti-GFP (1:5000, #ab13970, Abcam), anti-Kif21B 1:500, #NBP1-84188, Novus biologicals), anti-Kif13B (1:500, #SAB 2101257, Sigma-Aldrich), anti-Map2 (1:1000, #188004, Synaptic systems), anti-Piccolo (1:500, #142104, Synaptic systems) and anti-PSD95 (1:1000, #MA1-045, Thermo Fisher Scientific). After overnight incubation, cells were washed thoroughly, three rinses in PBS and the immunoreactivity was probed using Alexa 405/488/546/568-conjugated secondary antibodies (1:500; Molecular Probes) for 1 hr. at room temperature. After washing three times with PBS, the coverslips were mounted using Tris-fluoro gel. Images were acquired by using Zeiss LSM 780 confocal microscope system with 63X objective. Only projection images are shown and used for analysis. Student’s *t* test or one-way ANOVA was used for comparison between two groups, and statistical significance was defined as p < 0.05.

### Structured Illumination Super-Resolution Microscopy

Following immunocytochemistry, neurons were imaged using a Zeiss ELYRA PS.1 instrument (Carl Zeiss, Jena, Germany) at a resolution of 1028 by 1028 pixels, using a Zeiss 63X/1.4 NA Plan Apochromatic objective. Each fluorescent channel, 405, 488 and 561 were acquired using three pattern rotations with 3 translational shifts. The final SIM projection images were reconstructed using Zen 2013 (Carl Zeiss, Jena, Germany) and analyzed using ImageJ.

### Experimental Design and Statistical Analysis

Details of experimental design and analyses for experiments are described under each experiment sections. Schematics in each figure shows overall experimental design. The numbers of replications are indicated directly in the figure or in Figure legends section. Details of statistical tests and values used in the plots (electrophysiology, qPCR and imaging data) are described in the Dataset 1, Supplementary Table Additional Files.

### Ethics Approval and Consent to Participate

Animal experimental methodologies were reviewed and approved by the Institutional Animal Care and Use Committee of TSRI.

## Electronic supplementary material

SUPPLEMENTARY INFORMATION

Supplementary Dataset 1

## Data Availability

All data analyzed in this study are reported in the manuscript.
