# Supplementary material for: Kinesin Family of Proteins Kif11 and Kif21B Act as Inhibitory Constraints of Excitatory Synaptic Transmission Through Distinct Mechanisms
Source: Sci Rep. 2018 Nov 27;8:17419. doi: 10.1038/s41598-018-35634-7 (PMC6258692; doi:10.1038/s41598-018-35634-7)
Supplement: Supplementary file 1 — SUPPLEMENTARY INFORMATION [file 41598_2018_35634_MOESM1_ESM.pdf]

Kinesin Family of Proteins Kif11 and Kif21B Act as Inhibitory Constraints Of Excitatory Synaptic Transmission Through Distinct Mechanisms

Supriya Swarnkar[#], Yosef Avchalumov[#], Bindu L Raveendra, Eddie Grinman, Sathyanarayanan V Puthanveettil*

Supplementary Information

3 Supplementary data figures (S1, S2 and S3)

7 Additional file tables (S1-S7)

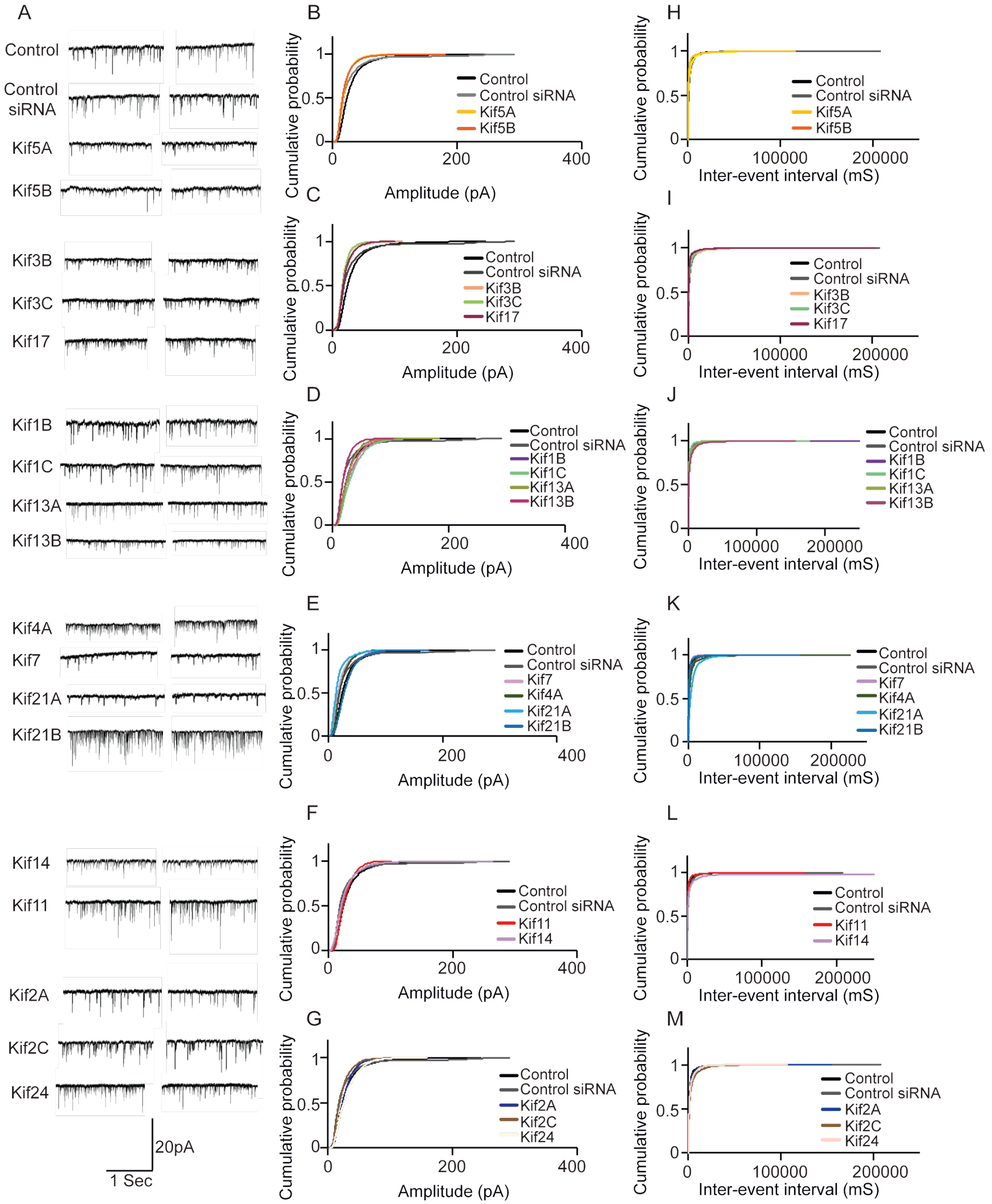

Supplementary Figure S1. Cumulative probability analysis of sEPSCs. Following individual knockdown of 18 Kifs, sEPSCs were measured (See Figure 1). **A.** Two representative sEPSCs traces of control (no siRNA), control siRNA

(nontargeting siRNA) and knockdown of individual Kifs. **B-G.** Cumulative probability plots showing amplitude of sEPSCs between control, control siRNA and Kif siRNA. **H-M.** Cumulative probability plot showing frequency of sEPSCs between control, control siRNA and Kif siRNA.

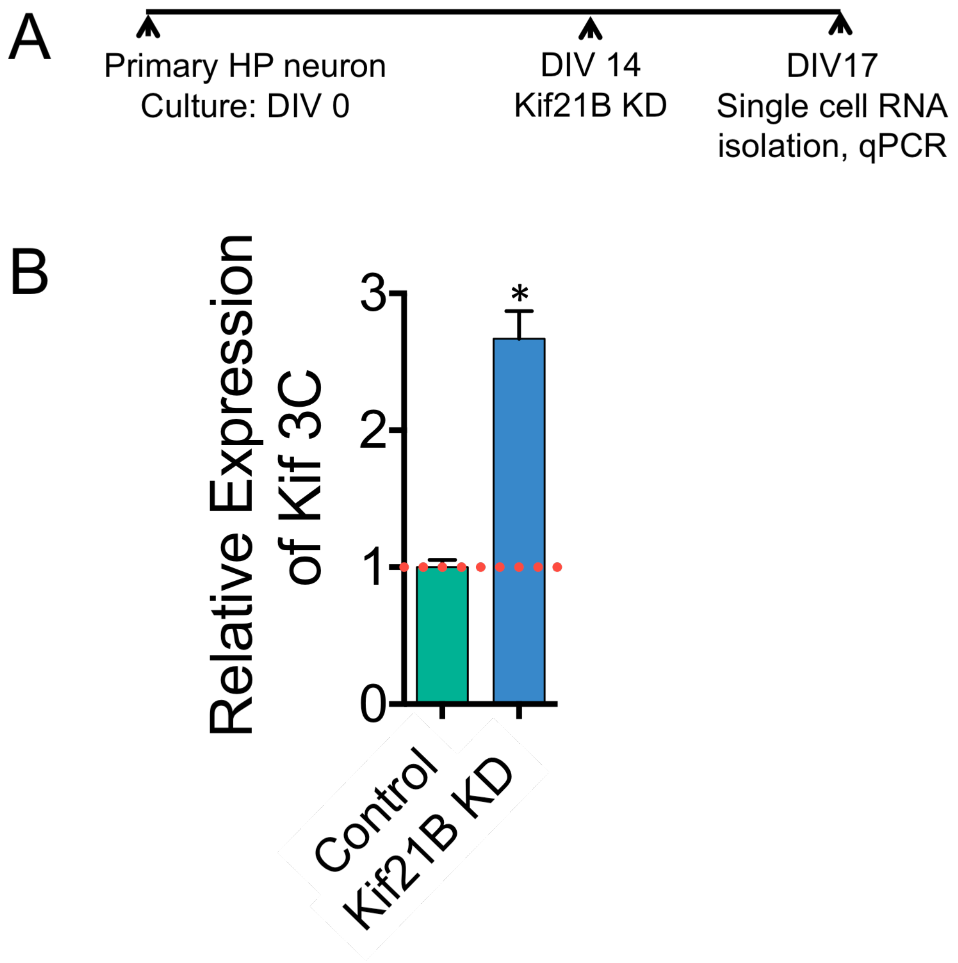

Supplementary Figure S2. Single cell qPCR analysis of expression of Kif3C following Kif21B knockdown. A. Schematics describing experimental time line. **B**. qPCR analyses of relative expression changes in Kif3C in Kif21B knockdown and control neurons. Kif21B shRNA or non-targeting shRNA plasmid co-expressing EGFP to identify transfected neurons were used. RNA from non-targeting shRNA transfected hippocampal neurons was used as control. Using Arcturus PicoPure RNA isolation kit (Thermo Fisher Scientific.), total RNAs were

isolated from the intracellular contents aspirated using a patch pipette. Number of GFP labeled neurons: 20 neurons per condition. Data was normalized to 18srRNA levels. Error bars are SEM, *p<0.05, Student's *t* test.

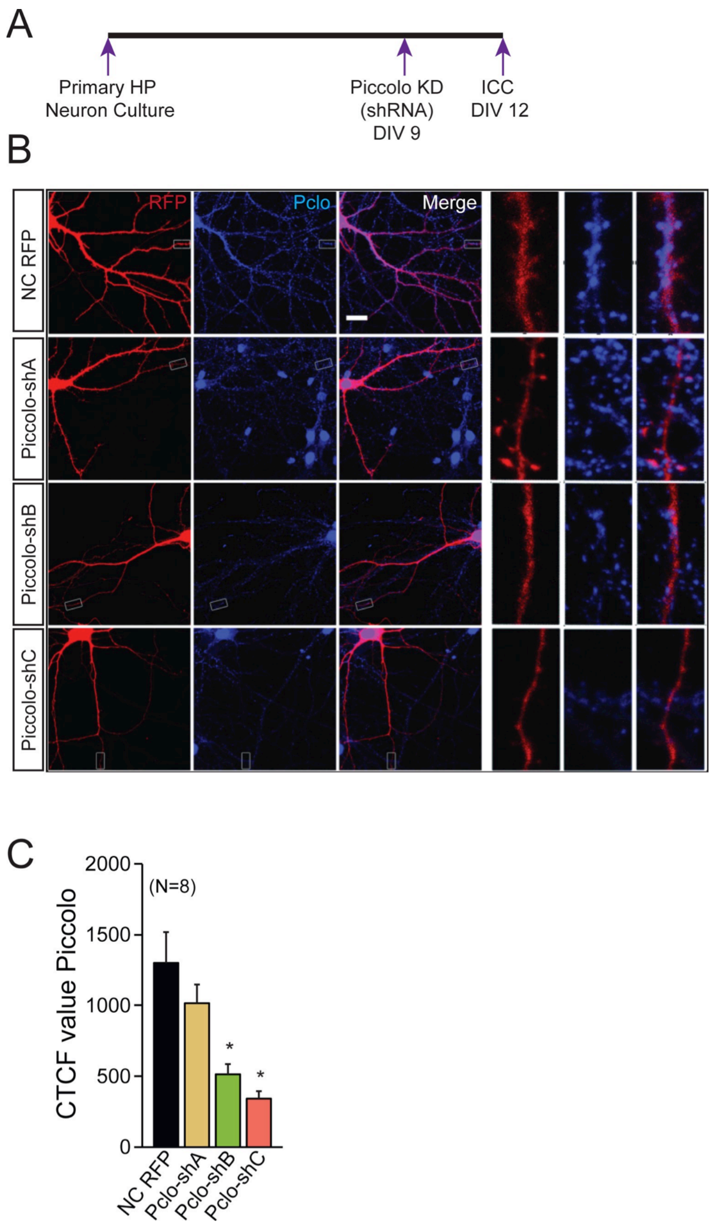

Supplementary Figure S3. Characterization of Piccolo knockdown. A. Schematic showing the experimental strategy. **B.** Confocal projection images showing Piccolo (Pclo) expression in hippocampal neurons following its knockdown using three different shRNAs. A scrambled non-targeting shRNA was

used as control. Piccolo in neurites show decrease in its expression 72 hrs. after transfection of shRNA plasmid expressing RFP (Pclo shB and shC) when compared to NC (non-targeting scrambled control) shRNA expressing RFP in cultured hippocampal neurons (DIV 12). RFP (red) and Piccolo (blue). Representative confocal projection images and digitally enlarged inset images are shown. **C**. Bar graph shows quantitative analyses (using NIH ImageJ) of corrected total cell fluorescence (CTCF) of Piccolo puncta (blue) in RFP (red) positive neurites after Piccolo knockdown using different shRNAs. Error bars are SEM. One-way ANOVA followed by Tukey's post hoc test. *$P<0.01$. Scale bar: 20µm. Also see Additional File Table S5 for values used in the bar graphs.

SUPPLEMENTARY TABLES

Additional file table legends:

Additional File supporting Figure 1 labeled as Table S1: qPCR analysis of Kif knockdown (KD) and sEPSC measurements following Kif knockdowns in hippocampal neurons are shown.

Additional File supporting Figure 2 labeled as Table S2: mEPSC measurements following Kif knockdowns in hippocampal neurons are shown.

Additional File supporting Figure 4 labeled as Table S3: Analysis of Kif expression following Kif11 and Kif21B knockdown are shown along with electrophysiological measurements to assess the necessity of Kif3C for Kif21B effects on synaptic transmission.

Additional File supporting Figure 5 labeled as Table S4: Assessments of pre-NMDAR function in mediating Kif11 effects on synaptic transmission.

Additional File supporting Figure 6 and Supplementary Figure S2 labeled as Table S5: Analysis of role of Kif11 in mediating structural changes in hippocampal neurons.

Additional File supporting Figure 7 labeled as Table S6: Necessity of Piccolo expression in mediating Kif11 effects on synaptic transmission.

Additional File labeled as Table S7: Sequences of oligonucleotide primers used in the gene expression analyses.
